# Coming up short: Comparing venous blood, dried blood spots & saliva samples for measuring telomere length in health equity research

**DOI:** 10.1371/journal.pone.0255237

**Published:** 2021-08-18

**Authors:** Arline T. Geronimus, John Bound, Colter Mitchell, Aresha Martinez-Cardoso, Linnea Evans, Landon Hughes, Lisa Schneper, Daniel A. Notterman

**Affiliations:** 1 School of Public Health, University of Michigan, Ann Arbor, Michigan, United States of America; 2 Population Studies Center, Institute for Social Research, University of Michigan, Ann Arbor, Michigan, United States of America; 3 National Academy of Medicine, Washington, DC, United States of America; 4 Department of Economics, University of Michigan, Ann Arbor, Michigan, United States of America; 5 Department of Public Health Sciences, University of Chicago, Chicago, Illinois, United States of America; 6 Center for Health Equity Research, Northern Arizona University, Flagstaff, Arizona, United States of America; 7 Department of Molecular Biology, Princeton University, Princeton, New Jersey, United States of America; University of California-Irvine, UNITED STATES

## Abstract

**Background:**

Telomere length (TL) in peripheral blood mononuclear cells (PBMC) from fresh venous blood is increasingly used to estimate molecular impacts of accumulated social adversity on population health. Sometimes, TL extracted from saliva or dried blood spots (DBS) are substituted as less invasive and more scalable specimen collection methods; yet, are they interchangeable with fresh blood? Studies find TL is correlated across tissues, but have not addressed the critical question for social epidemiological applications: Do different specimen types show the same association between TL and social constructs?

**Methods:**

We integrate expertise in social epidemiology, molecular biology, and the statistical impact of measurement error on parameter estimates. Recruiting a diverse sample of 132 Metro-Detroit women, we measure TL for each woman from fresh blood PBMC, DBS, and saliva. Using regression methods, we estimate associations between social characteristics and TL, comparing estimates across specimen types for each woman.

**Results:**

Associations between TL and social characteristics vary by specimen type collected from the same woman, sometimes qualitatively altering estimates of the magnitude or direction of a theorized relationship. Being Black is associated with shorter TL in PBMC, but longer TL in saliva or DBS. Education is positively associated with TL in fresh blood, but negatively associated with TL using DBS.

**Conclusion:**

Findings raise concerns about the use of TL measures derived from different tissues in social epidemiological research. Investigators need to consider the possibility that associations between social variables and TL may be systematically related to specimen type, rather than be valid indicators of socially-patterned biopsychosocial processes.

## Introduction

Telomeres, the protein and DNA caps on chromosomes, shorten with cell division until a point at which cellular senescence, death, or mutation results. As such, the average length of telomeres reflects the replicative history of the analyzed cell lineages [1]. A growing number of investigators across scientific fields have theorized and begun to empirically study the role of telomeres in health and aging, either in terms of explicating their properties as functional mechanisms of disease and aging, or as “sentinels” for environmental exposures, psychosocial stressors and resultant population inequities in health over the life course [2–4]. Investigators interested in the latter use, interpret TL as a proxy for the physiologic impacts of accumulated life experience or weathering [4–11]. Evidence that TL may be an indicator of stress-mediated biological aging comes from several sources [3, 5, 12–16]. TL is not a direct marker of stress; however, increased cellular division—perhaps due to inflammation or immune activation, or stress-induced repression of stem cell telomerase activity—can accentuate the loss of telomere sequence. In the social epidemiological context, TL may be of predictive value, whether or not functional mechanisms are perfectly specified.

While telomere length varies across tissues, measuring TL via leukocyte-derived DNA extracted from fresh venous blood (i.e., peripheral blood mononuclear or PBMC) samples constitutes a widely accepted approach in studies of biopsychosocial stressors and health. TL measured from PBMC samples (as well as whole venous blood) has been commonly linked to morbidity and mortality in the human literature [17–20]. It is also possible that PBMCs, by virtue of representing a population of immune cells, provide a sharper and more responsive indicator of stress-related chronic inflammation and exposure to infectious disease than alternatives. However, the process of measuring TL PBMCs extracted by fresh venous blood is expensive, time-consuming, invasive, and requires specialized clinical expertise, lab equipment, and handling at the collection site. Consequently, it is inaccessible in remote areas, can adversely affect the recruitment and retention of hard-to-reach or mistrustful research participants; as well as being costly for studies of large populations, hampering variation in social experience reflected in or the representativeness of samples. This can pose a major logistical problem for observational studies of population health and aging inequity, which require diverse samples that include the most marginalized members of society [21].

Increasingly, investigators examining TL in large, population-based studies have turned to measurement of telomere length in DNA derived from saliva or buccal swabs [22–26] and some researchers have begun to include telomere data drawn from finger-prick dried blood spots (DBS) [27–29]. These approaches are seen as minimally invasive, scalable, and less expensive alternatives to venous blood that could be used widely in social epidemiologic and population health studies, including in hard-to-reach populations. However, as outlined below for each, respectively, using TL measures derived from DNA extracted from saliva or DBS in population-level studies could be questioned.

While there is no *a priori* basis to prefer one sample type over the other, the cellular composition of saliva and peripheral blood differs, and some of these cellular constituents offer diverging replicative histories. Therefore, there is reason to expect that the TL of saliva and blood-derived samples may diverge, and that the extent of divergence may vary in any particular individual due to changes in cellular composition, environment, or health status. Of greatest importance, if this divergence were systematically related to factors central to social research, such as measures of structured life stressors, socioeconomic position, or ethnic/racial identity, this would represent a serious challenge to using TL from saliva to measure socially patterned group differences in biological health and aging.

DBS has been used for newborn screening and has, over the last two decades, been adapted to a wide range of metabolic, immunologic, and genetic disorders in newborns and is sometimes stored in big data sets [30–32]. While drawing on the same tissue (blood) as using venous collection techniques, as with saliva, there is still reason to be skeptical of the use of DBS for measuring TL given the limitations of DBS technology, including the use of filter paper that is neither optimized to support DNA samples nor optimized for long-term storage. This is compounded by the diverse, and often adverse, storage conditions of DBS.

Previous studies comparing TL in matching DBS or saliva and venous whole blood have found that while correlative, the mean TL differs [28, 29, 33, 34]. A recent study examining relative TL correlation between 41 tissue pairs of samples including more than 20 tissue types; with each an average of seven different tissue TL measurements per donor, showed general positive correlations which differed in different tissues [35]. As expected, the highest correlation was between tissue pairs from the same organ (e.g. sigmoid and transverse colon) followed by higher correlation between tissues having common development origins (thyroid and brain cerebellum; mesodermal and ectodermal origin; or endodermal origin). Age, BMI, age-related chronic disease status, smoking status, and rare, loss of function variants in telomere maintenance genes were negatively associated with TL and African ancestry was associated with longer TL compared with European ancestry across non-reproductive tissue types in this select donor sample [35]. Interestingly, higher correlation between tissue types was observed between individuals with smoking history compared with never-smokers [35], although the authors did not provide an explanation; however, smoking has been previously associated with shorter telomeres [36]. That finding may be of relevance to this study, since smoking prevalence is socially patterned and, thus, likely to reference unobserved social characteristics as well as the direct impact of smoking, itself.

The fact that TL measures taken from different specimens are correlated is insufficient to deem them interchangeable substitutes in studies of population health inequity. A theoretical precondition for using TL measures derived from different specimens in studies intended to describe and test relationships between social conditions and health outcomes is that associations based on various TL measures reflect these social conditions in similar ways. For example, if using TL as an indicator of whether systemic racism subjects US Black people to greater physiological wear and tear at earlier ages than US White people, it would be essential that investigators would arrive at the same answer on whether Black people of the same chronological age have shorter or longer TL than White people without regard to specimen type (e.g., blood or saliva) or method of obtaining the specimen (e.g., venous blood draw or finger prick blood spot). Differences in cellular replication rates across tissues are well described and are both intrinsic to a tissue and may also reflect environmental inputs [35]. This suggests that differing average TL across tissues could, in part, reflect environmental or ancestry inputs that vary by unobserved factors associated with structured differences in lived experience [5]. This study considers this critical question.

The primary question is whether the error introduced when using a given measure of TL as an indicator of socially triggered biopsychosocial effects is systematic with respect to populations of interest. For example, one might be interested in estimating racial/ethnic or socioeconomic differences in biological age (conditional on chronological age) using measured TL as the indicator of biological age, where “biological age” is a construct used to denote a variety of adverse physiological effects often associated with aging. If the error is systematic with respect to race or socioeconomic position, the estimates using TL could, systematically over- or under- estimate actual differences in biological age.

Since “biological age” is a construct, we cannot directly test for such systematic differences—i.e., there is no gold standard for biological age. However, we can test to see whether there is evidence of systematic differences that *vary by specimen type*. If measured TL is a valid indicator of biological age, and if this is equally true independent of the specimen types used to measure TL, then we would expect TL differences between groups to be similar independent of specimen type used.

If there is a systematic error, then whether the population differences in TL were estimated from saliva, DBS, or venous blood would be of consequence. Specimen choice could potentially lead to differing conclusions about whether, in which direction, and of what magnitude TL of various populations differ depending on the tissue and technology used. If the measurement error is random with respect to population group, however, then inferences of population differences in TL based on saliva or DBS would be valid. By validating the use in population-level studies of TL measures derived from saliva or DBS compared to fresh blood, we hope to inform interpretation of existing studies using DBS or saliva collections and evaluate saliva or DBS as potential alternatives to venous blood collection for future studies.

## Methods

We recruited a total of 132 non-Hispanic Black, non-Hispanic White, and Mexican-descent Michigan women residents from high-poverty areas of Detroit and a more affluent area outside of Detroit, Ann Arbor, MI, ages 25–49. TL was measured in DNA from three distinct specimen types from each woman: fresh blood (PBMC), dried blood spot (DBS), and saliva, collected during the same study appointment. We were able to analyze a convenience sample because our primary scientific question is whether *within-woman* TL differences found in fresh blood cells compared to DBS or to saliva are systematic or random with respect to age, race/ethnicity, education, or residential area − and not whether there are racial/ethnic or other differences in TL, *per se*. Theoretically, if telomere length is an indicator of a woman’s biological age relative to another woman, contrasts should be independent of the tissue type used whether or not the women being chosen are a random sample of a larger universe.

The research protocol was approved by the University of Michigan Institutional Review Board. Details of the recruitment and specimen collection methods are provided in the S1 Appendix. In brief, Detroit, MI participants were recruited through paper flyers we distributed across the city (bus stops, beauty shops, laundromats, grocery markets, apartment complexes) and at community events (swap meets, community yard sales, sports events and concerts, and summer festivals), as well as on social media (e.g., Facebook community groups and non-profit organization sites specific to Detroit). The flyer, written in both English and Spanish, described the study and listed a telephone number for more information. We recruited participants from the Ann Arbor, MI area using a university-administered website, UM Clinical Studies, which connects potential volunteers with researchers. Additionally, we posted flyers across campus inviting eligible women to contact our study personnel for further information.

When potential participants called for more information, our project staff described the study purpose and protocol, completed a standardized screener brief intake to assess participant eligibility, answered questions and then scheduled an appointment if they agreed to participate in the study. Participants chose whether they preferred to have the data collection visit conducted in English or Spanish and, in Detroit, whether they preferred to have their specimens collected through a home visit or at the University of Michigan Detroit Center, a UM building in Detroit that accommodates research projects and outreach initiatives. Data collection in Ann Arbor occurred in a private room at the University of Michigan Institute for Social Research.

Three ethnically diverse teams obtained written informed consent and collected the data. Each team included a team leader, their assistant, and a phlebotomist; each team included a native Spanish speaker. The teams were led by a Mexican-descent woman, an African American woman, and an Arab-American woman, representing the three major racial/ethnic groups in Detroit. The phlebotomists were current Detroit residents, a Black male, a Black female, and a White female.

All specimens were prepared for shipment in the same UM lab and by the same technician; after shipment all were analyzed in the same lab at Princeton University. See S1 Appendix for full details of telomere measurement. We note here that batch effects can be an important source of bias [37]. Batch effects can occur when groups of samples are measured under different conditions as when a large collection of samples is measured over several days in different tranches. To minimize batch effects when measuring telomere length in this study, all samples from the same individual were included on the same qPCR run. Standard (serial dilutions of double stranded oligonucleotides), primers and control DNA templates (genomic DNA samples included in every run) were diluted to appropriate concentrations in sufficient quantities for the entire project, aliquoted for single use and stored at -80°C. The same lot of Quantitect SybrGreen PCR kit (Qiagen, Hilden Germany), was used for the entire project. Upon thawing, the SybrGreen was aliquoted for single use and stored at -20°C in the dark. In addition, each PCR plate contained several control samples that were replicated across all plates. This allowed for detection and adjustment of residual plate-to-plate measurement differences. To minimize residual bias introduced by batch effects, the values were adjusted (“normalized”) by a term derived from the concurrently measured telomere mass or 36B4 mass of repeat samples introduced into each PCR plate [38].

## Statistical analysis

Telomere and 36B4 quantities were interpolated from the standard curve generated from the reference fragments of known length. All samples were divided into three technical replicates, randomly distributed in the plate, and averaged in the following formula:

ln(TL)=[(Avg.ln(Telqty)−ln(Telnormalizationfactor))]−[(Avg.ln(36B4Qty))−ln(36B4normalizationfactor))]−ln(92) (1)

Note that (1) represents ln(TL) as linear in terms. This turns out to be useful from the point of view of our statistical analysis. The standard formulation would have used:

ln([(Avg.TelQty/Telnormalizationfactor)/(Avg.36B4Quantity/36B4normalizationfactor)]/92)[26,33], (2)

rather than the expression in (1), which is not linear in terms. In our samples the correlation between quantity (1) and (2) is very high (above 0.999) and, not surprisingly, using (1) rather than (2) has negligible effects on our results. Both expressions relate the detected mass of telomeric DNA to the mass of another gene (*36B4*), which has been shown to be a stable reference gene.

We first compared the distribution of ln(TL) derived from the three specimens. We compared means and standard deviations, and examined the correlations of ln(TL) derived from the three specimens. As discussed above, much of the interest in TL involves using measured TL as an indicator of what has been termed, “biological age.” What researchers are typically interested in is the association between biological age (conditional on chronological age) and various aspects of individuals’ socially structured experience. Thus, for example, researchers have been interested in the effects of stress on telomere length [13, 19] in the association between race and telomere length [5], and in the effect of early disadvantage on telomere length [33]. Thinking of measured TL as a proxy for biological age, researchers run regressions of the form:

ln(TL)=X’β+μ, (3)

where *X* includes both variables of particular interest and controls. While we cannot directly test the validity of measured ln(*TL*) as a proxy for biological age, we can compare estimates of β across specimen types. If measured ln(*TL*) is a valid measure of biological age for all specimen types then estimates of β should not vary significantly across specimen types. If, on the other hand, estimates of β vary significantly, this calls into question this assumption. A formalized version of this argument can be found in the S1 Appendix.

With this framework in mind, we regressed our ln(*TL*) measures on the age of our respondents, dummies representing their race and ethnicity, their educational attainment and a location dummy (Ann Arbor vs Detroit). We collected measures on these because we expect such factors to have an independent correlation with biological age and to be well measured. Since our samples are convenience samples and do not follow individuals over time, one cannot interpret the coefficients as reflecting cross group differences for anything but this sample. However, if TL measures based on the different tissues are all valid measures of biological age we would expect the association between age, race, place or education and TL to be the same across these different specimens within the precision of the assay.

## Results

Table 1 lists the sample’s demographic characteristics. Table 2 shows the number of usable TL measures by specimen type. Of our DBS samples, 33 had insufficient DNA to analyze. Table 3 compares characteristics of the samples where we were and were not able to calculate TL based on the DBS data. Those with missing TL measures based on DBS were more likely to be from Ann Arbor, to be white and to have a college education. Conditional on covariates, we found no evidence of differences in ln (TL) between the samples with and without usable DBS data. There is no reason to believe that the fact that DBS was missing for a third of the overall sample invalidates the within woman comparisons in existing sample.

**Table 1:** Characteristics of Ann Arbor and Detroit participants.

	Share	
	Ann Arbor	Detroit
Non-Hispanic White	0.41	0.35
Non-Hispanic Black	0.35	0.32
Hispanic	0.25	0.34
High School Graduate*	0.99	0.83
College Graduate	0.78	0.22
Age 35+	0.62	0.41
N	69	63

Note: Share high school graduates exclusive of college graduates

**Table 2:** Number of valid TL measures by specimen Type.

	Sample				
Number of Measures	With DBS available			Full Sample	
	PBMC	Saliva	DBS	PBMC	Saliva
2	28	20	21	37	26
3	71	79	78	95	106
Total	99	99	99	132	132

**Table 3:** Comparing samples with and without DBS data.

	Sample	
	With DBS Available	Full Sample
	Share	Share
Detroit	0.55	0.48
Non-Hispanic White	0.33	0.38
Non-Hispanic Black	0.33	0.33
Hispanic	0.34	0.29
High School Graduate*	0.41	0.39
College Graduate	0.47	0.51
Age 35+	0.53	0.51
N	99	132

Note: Share high school graduates exclusive of college graduates

Table 4 reports means and standard deviations for ln(TL) for each specimen type as measured above, together with the correlations across the measures for the two samples. To aid in the interpretation of these numbers we report both mean ln(TL) and exp[mean ln(TL)]. Measured average ln(TL) is somewhat larger based on the fresh blood specimens than it is using the saliva specimens, though the difference is small and statistically insignificant. In the sample that includes the DBS specimens, average ln(TL) based on the DBS specimen is somewhat larger than is average ln(TL) based on the fresh blood specimens. The variance ln(TL) also fluctuates a bit across the specimens, though, again, the observed differences are not statistically significant. All three sample types provided average TL that are appropriate for individuals in the age range of 25–49 years (for PBMC, 7.31 kb).

**Table 4:** Mean, standard deviations, and correlations of ln(TL) by specimen type.

		Exponentiated Mean TL			Correlations		
Sample	Specimen		Mean	Std Dev	PBMC	Saliva	DBS
Full Sample	PBMC	7.36	2.00	0.22	1.00		
	Saliva	6.48	1.87	0.21	0.59	1.00	
Sample with DBS Available	PBMC	7.28	1.99	0.23	1.00		
	Saliva	6.48	1.87	0.21	0.62	1.00	
	DBS	7.85	2.06	0.26	0.57	0.52	1.00

**Fig 1:**
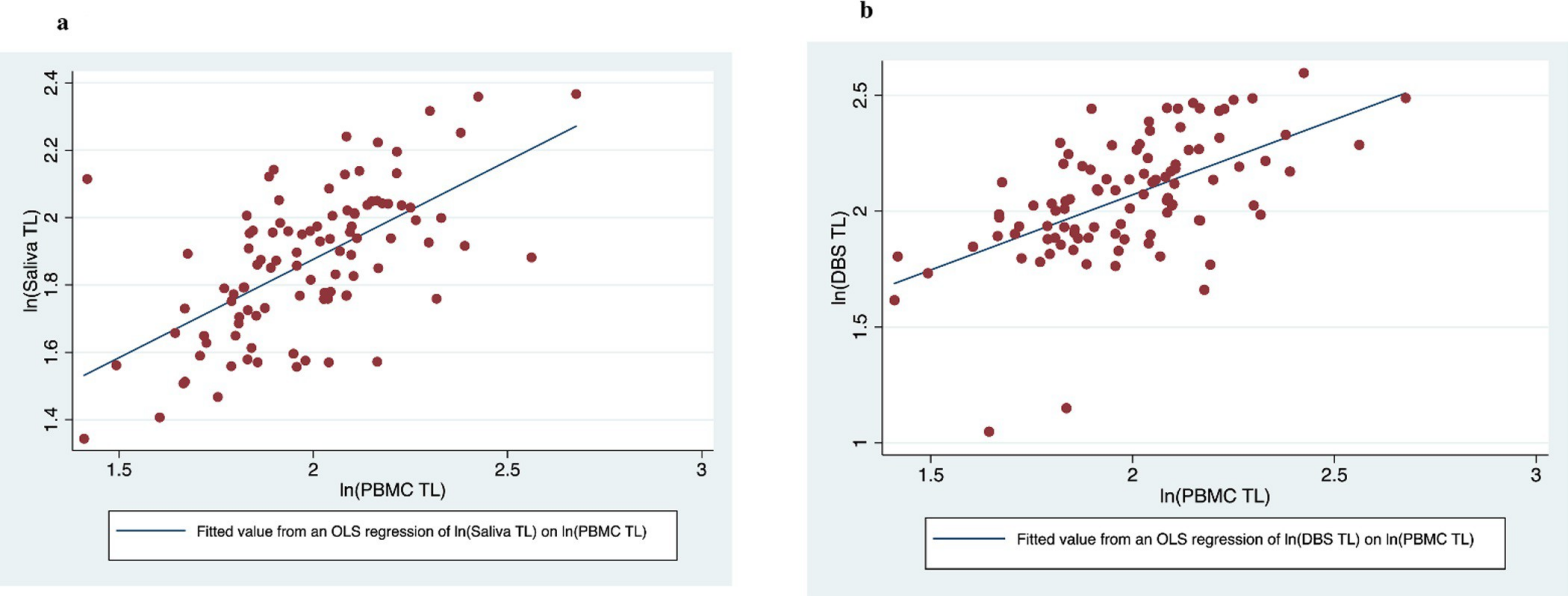
a. Salivary Telomere Length vs. PBMC Telomere Length. b. DBS Telomere Length v. PBMC Telomere Length.

How highly correlated are our ln(TL) measures drawn from different specimen types? Looking back at Table 4 we see the correlation between ln(TL) measured using the fresh blood and saliva specimens tends to be around 0.60, while that between ln(TL) measured using the dried blood spot specimen and the fresh blood or saliva samples is a bit lower (0.57 and 0.52, respectively). Fig 1A and 1B graphically depict the association between ln(TL) measured in various specimens and ln(TL) measured using the fresh blood sample.

In Table 5 we report results from ordinary least squares regressions of the ln(TL) measures on age, race/ethnicity, education and location dummies by specimen type drawn from the same woman. Race and ethnicity, educational attainment and location were all defined in terms of exclusive categories with white, high school dropout and Ann Arbor representing the left out categories. Age was defined linearly in terms of single year of age divided by 10. Using age/10 rather than age simply rescaled the estimated coefficient.

**Table 5:** The estimated effect of age, location, race, and education on ln(TL) by specimen type.

	Full Sample			Sample with DBS Available				
	1	2	3	4	5	6	7	8
Dep.Variable	PBMC	Saliva	Saliva-PBMC	PBMC	Saliva	Saliva-PBMC	DBS	DBS-PBMC
Age/10	-0.051	0.016	0.067	-0.069	-0.031	0.038	-0.045	0.023
	(0.026)	(0.026)	(0.022)	(0.031)	(0.029)	(0.028)	(0.043)	(0.036)
Detroit	0.067	0.031	0.036	0.089	0.029	0.060	0.109	-0.020
	(0.048)	(0.043)	(0.042)	(0.055)	(0.053)	(0.048)	(0.074)	(0.061)
Black	-0.031	0.071	0.102	-0.028	0.076	0.104	0.064	0.092
	(0.049)	(0.044)	(0.040)	(0.056)	(0.054)	(0.047)	(0.067)	(0.056)
Hispanic	-0.022	-0.002	0.020	0.025	0.026	0.001	0.044	0.019
	(0.043)	(0.044)	(0.039)	(0.050)	(0.052)	(0.045)	(0.067)	(0.056)
High School	0.045	0.044	-0.001	0.066	0.06	-0.006	-0.137	-0.203
Graduate	(0.061)	(0.073)	(0.049)	(0.058)	(0.073)	(0.053)	(0.080)	(0.059)
College Graduate	0.067	0.057	-0.011	0.116	0.074	-0.042	-0.018	-0.134
	(0.063)	(0.076)	(0.053)	(0.060)	(0.78)	(0.056)	(0.081)	(0.064)
Joint Test#			0.004			0.086		0.012

Robust standard errors reported in parenthesis [39]. # P-value for joint significance of the coefficients reported in column (3), (6) and (8).

In the left panels of the table, we show coefficient estimates comparing results using alternatively the PBMC and saliva measures of TL using the full sample (N = 132). In the right-hand panels, we report comparisons between coefficients using the PBMC, Saliva and DBS measures using the smaller sample (N = 99) that includes TL measures derived from DBS. Note that because different samples are used, the point estimates using the PBMC and saliva measures vary a bit between the two samples. In columns (3), (6) and (8) we report the differences between results based on the Saliva and DBS specimens vs the PBMC specimens. Thus, for example, the first estimate in the column, 0.067 equals 0.016 minus -0.051, the coefficient estimates reported in column 2 and 1 respectively. Statistical tests of the differences between the coefficients reported in columns (1) and (2), columns (4) and (5) and columns (7) and (8) amounts to a test of the joint statistical significance of the coefficients in columns (3), (6) and (8). P-values for these tests are reported at the bottom of the table.

We are primarily interested in whether estimated coefficients differ by specimen type. Depending on the samples, we can reject the equality of coefficients between the PBMC and Saliva tissues at the 0.004 or the 0.086 levels of significance and between the PBMC and the DBS tissues at the 0.012 level of significance. Looking at the estimated coefficient, we see the difference between the estimated effect of either age or being Black on our measures of ln(TL) varies significantly between the two different specimen types, including in the direction of the association. For example, using PBMC to measure TL, we find being Black is associated with shorter TL; while using saliva to measure TL, being Black is associated with longer TL in the same sample of the same women. Turning to DBS, we find, once again, that analyses involving PBMC specimens suggest Black people have shorter telomeres, while analyses involving DBS specimens suggest Black people have longer telomeres in the same sample. In the comparisons with DBS, we also find that the associations between education variables and TL go in different directions depending on specimen type. One would infer that education was positively associated with TL using PBMC, but that it was negatively associated with TL using DBS in the same sample.

The S1 Appendix includes results from analysis of samples in which we deleted observations with Cook’s D above 1 from the analysis. Our results are not sensitive to the deletion of outliers from our samples. The deletion of such observations does not qualitatively change our conclusion and, if anything, increases our confidence in the statistical significance of the differences between estimated coefficients.

## Discussion

If population researchers are to use TL measures based on saliva or DBS samples, we would hope that TL measures based on such specimens would be correlated with measures based on fresh blood samples, *and* would show similar contrasts between different population groups. In this sample the association between age and TL was stronger when we used fresh blood than when we used the other specimens (saliva or DBS), and the association between being Black (relative to White) and TL reversed sign depending on the specimen type used. Overall, study results suggest that what a researcher might conclude about differences in telomere length across age, racial/ethnic or educational groups could vary depending on specimen type, sample collection, processing, and storage methods.

As noted earlier, different tissues and their associated cell lineages have dissimilar replication histories that are both intrinsic to a tissue and may reflect environmental inputs [35]. These could result in differing average telomere lengths across tissues, differences that could vary with age or other characteristics of individuals. While this interpretation might account for the observed differences, the practical implication of our results remains the same: social epidemiological researchers using TL as an indicator of biological age might come to qualitatively different conclusions depending on whether the measures were derived from fresh blood, dried blood or saliva.

It should not be a surprise that the correlation between ln(TL) measured using fresh blood versus saliva is < 1 because the DNA in cells from different tissues will have different replicative histories even in the same woman; however, the fact that the correlation between ln(TL) measured using venous blood and DBS from the same blood is < 1 and lower than the correlation between venous blood and saliva is of concern and indicates that more research is necessary to define factors that affect the accuracy of TL measurements in DBS. The need for this research is more salient when investigators consider the use of lengthy storage of DBS, for example from a newborn testing program, rather than recently prepared DBS as used in this study, since the effect of storage condition is likely to be important and as yet not well characterized.

### Limitations and robustness check

By using women as their own controls and by measuring TL in different specimens for the same woman in the same batch, in the same lab, we were able to reduce important confounds in telomere length measurement and increase the efficiency of our sample. Our reliability estimates were 0.98 showing great consistency in measurement by the lab (see S1 Appendix for details). There are also questions in the literature about whether results from different labs or using different procedures can be compared; this study does not speak to those questions.

The belief that any individual can draw finger prick blood spots for TL measurement is a presumed practical benefit of using DBS over venous blood where a trained phlebotomist is needed. Yet, a sizeable share of our DBS specimens had insufficient DNA for telomere measurement. This suggests the critical importance of collecting an ample blood spot from each research participant. For us, the loss of a portion of DBS sample decreased sample size for comparisons of TL measured from DBS to other specimens. Since our study focused within-woman, the loss of DBS sample did not invalidate the study conclusions. However, for the more typical study of population differences in TL, if the investigator were relying on DBS alone to measure TL, loss of a sizeable portion of the sample would raise questions about the representativeness of the remaining sample and the generalizability of conclusions drawn. This suggests that it will be essential to assure adequate sample size when estimating DNA from DBS and may be a limitation of using DBS in population health equity studies.

The saliva was purified using a salting out method, whereas the venous PBMC and finger-pricked DBS samples were purified using PureLink kits, which use a silica-based column. The methods used were chosen based upon popular purification methods for each sample type. Using different DNA extraction methods can be a confounder of accurate TL measurements [40–43]. Notably, even though the PureLink kit was used for both the PBMC and DBS samples, preprocessing of the DBS prior to addition to the column differs from that of the PBMC due to the inherent differences between the samples. While ours is the first study to compare these particular methods of purification with respect to absolute TL, previous studies have not shown a difference in relative TL between genomic DNA isolated from whole blood using a high salt extraction method [44] and the PureLink Kit [42]. Our results are consistent with findings of longer TL in finger-prick DBS samples compared with venous blood and longer TL in PBMC samples compared with Oragene saliva [34]. These findings contradict others [28], thus further replication by other labs is warranted.

Another potential bench concern may be that specific anticoagulant employed, heparin, has been shown in some studies to affect qPCR reactions [45], and so may add noise to our TL measurements. As a robustness check, we found that heparinase pretreatment of our purified DNA did not result in increased TL, although heparinase pretreatment did restore TL in other heparin-treated samples prepared in the laboratory. Thus, we confirmed experimentally that the TL measurements in these samples were not affected by the use of heparin as the anticoagulant. Details and results of this experiment are provided in the S1 Appendix.

## Summary and conclusion

We undertook a primary data collection effort where we prioritized including racially diverse sample members including those residing in disinvested high poverty areas because of our interest in applying lessons learned to population health equity research in hard-to-reach populations, where the logistical practicalities of collecting DBS or saliva might outweigh any greater confidence one might have in fresh venous blood samples. Study findings should be replicated in other samples. However, we find evidence that the magnitude and sign of the association between TL and age, race, and education varies according to specimen type from the same woman. These findings raise significant concerns about the uncritical use of TL measures derived from diverse sources such as blood PBMC, saliva or dried blood in health equity research without further intensive research to determine the sources of variation and their effects.

The promise of including TL variables in health equity research lies in their potential to identify the biopsychosocial pathways through which social inequity impacts population health. Yet, social research enlisting biological variables is historically fraught. Understanding what such variables measure and the realm of appropriate interpretations of their associations with social variables requires care. This suggests the importance of truly collaborative research partnerships between biological and social science researchers when addressing questions of possible molecular pathways related to population health equity, including arriving at conceptual clarity regarding the biopsychosocial hypotheses being tested and the statistical impact of measurement error that may be relevant to assess [46]. Knowing only that TL is correlated across tissues is insufficient.

Based on our findings we would caution investigators and consumers of the extant literature measuring TL using DBS or saliva to be appropriately qualified in their conclusions relative to studies using fresh blood. In addition, more basic research needs to be done to facilitate our understanding of how saliva and blood-based measurement of TL should be reconciled. Our findings underscore the need for researchers estimating differences in TL across socially salient population groups—whatever specimen or data collection method used–to be exceedingly cautious when making *post hoc* interpretations of differences found–for example between racial/ethnic or socioeconomic groups–as these may be systematically related to specimen type, rather than be valid indicators of biopsychosocial processes.

## Supporting information

S1 Appendix Supplementary information. (DOCX)

## References

[1] Notterman DA, Schneper L. Telomere Time—Why We Should Treat Biological Age Cautiously. JAMA Netw Open. 2020;3: e204352. doi: 10.1001/jamanetworkopen.2020.4352 32364591

[2] National Institute of Environmental Health Sciences and National Institute on Aging. Telomeres as Sentinels for Environmental Exposures, Psychosocial Stress, and Disease Susceptibility. 2017.

[3] Allsopp R. Take a Ride on the Telomere-Aging Train. The Journals of Gerontology: Series A. 2021;76: 1–2. doi: 10.1093/gerona/glaa245 33355657

[4] Puterman E, Gemmill A, Karasek D, Weir D, Adler NE, Prather AA, et al. Lifespan adversity and later adulthood telomere length in the nationally representative US Health and Retirement Study. Proc Natl Acad Sci USA. 2016;113: E6335–E6342. doi: 10.1073/pnas.1525602113 27698131 PMC5081642

[5] Geronimus AT, Pearson JA, Linnenbringer E, Schulz AJ, Reyes AG, Epel ES, et al. Race-Ethnicity, Poverty, Urban Stressors, and Telomere Length in a Detroit Community-based Sample. J Health Soc Behav. 2015;56: 199–224. doi: 10.1177/0022146515582100 25930147 PMC4621968

[6] Lu D, Palmer JR, Rosenberg L, Shields AE, Orr EH, DeVivo I, et al. Perceived racism in relation to telomere length among African American women in the Black Women’s Health Study. Ann Epidemiol. 2019;36: 33–39. doi: 10.1016/j.annepidem.2019.06.003 31387775 PMC7048405

[7] Chae DH, Epel ES, Nuru-Jeter AM, Lincoln KD, Taylor RJ, Lin J, et al. Discrimination, mental health, and leukocyte telomere length among African American men. Psychoneuroendocrinology. 2016;63: 10–16. doi: 10.1016/j.psyneuen.2015.09.001 26398001 PMC5407686

[8] Lucas T, Pierce J, Lumley MA, Granger DA, Lin J, Epel ES. Telomere length and procedural justice predict stress reactivity responses to unfair outcomes in African Americans. Psychoneuroendocrinology. 2017;86: 104–109. doi: 10.1016/j.psyneuen.2017.09.008 28938175

[9] McFarland MJ, Taylor J, McFarland CAS, Friedman KL. Perceived unfair treatment by police, race, and telomere length: a Nashville community-based sample of black and white men. J Health Soc Behav. 2018;59: 585–600. doi: 10.1177/0022146518811144 30417689

[10] Pantesco EJ, Leibel DK, Ashe JJ, Waldstein SR, Katzel LI, Liu HB, et al. Multiple forms of discrimination, social status, and telomere length: Interactions within race. Psychoneuroendocrinology. 2018;98: 119–126. doi: 10.1016/j.psyneuen.2018.08.012 30138832 PMC6359723

[11] Ruiz RJ, Trzeciakowski J, Moore T, Ayers KS, Pickler RH. Acculturation predicts negative affect and shortened telomere length. Biol Res Nurs. 2017;19: 28–35. doi: 10.1177/1099800416672005 27733476 PMC5942511

[12] Frenck RW, Blackburn EH, Shannon KM. The rate of telomere sequence loss in human leukocytes varies with age. Proc Natl Acad Sci U S A. 1998;95: 5607–5610. doi: 10.1073/pnas.95.10.5607 9576930 PMC20425

[13] Epel ES, Blackburn EH, Lin J, Dhabhar FS, Adler NE, Morrow JD, et al. Accelerated telomere shortening in response to life stress. Proc Natl Acad Sci U S A. 2004/12/01 ed. 2004;101: 17312–5. doi: 10.1073/pnas.0407162101 15574496 PMC534658

[14] Epel ES, Lithgow GJ. Stress Biology and Aging Mechanisms: Toward Understanding the Deep Connection Between Adaptation to Stress and Longevity. The Journals of Gerontology Series A: Biological Sciences and Medical Sciences. 2014;69: S10–S16. doi: 10.1093/gerona/glu055 24833580 PMC4022128

[15] Blackburn EH, Epel ES. Too toxic to ignore. Nature. 2012;490: 169–171. doi: 10.1038/490169a 23060172

[16] Allsopp RC, Vaziri H, Patterson C, Goldstein S, Younglai EV, Futcher AB, et al. Telomere length predicts replicative capacity of human fibroblasts. Proc Natl Acad Sci USA. 1992;89: 10114–10118. doi: 10.1073/pnas.89.21.10114 1438199 PMC50288

[17] Damjanovic AK, Yang Y, Glaser R, Kiecolt-Glaser JK, Nguyen H, Laskowski B, et al. Accelerated telomere erosion is associated with a declining immune function of caregivers of Alzheimer’s disease patients. J Immunol. 2007;179: 4249–4254. doi: 10.4049/jimmunol.179.6.4249 17785865 PMC2262924

[18] Tedone E, Arosio B, Colombo F, Ferri E, Asselineau D, Piette F, et al. Leukocyte Telomere Length in Alzheimer’s Disease Patients with a Different Rate of Progression. J Alzheimers Dis. 2015;46: 761–769. doi: 10.3233/JAD-142808 26402514

[19] Cherkas LF, Aviv A, Valdes AM, Hunkin JL, Gardner JP, Surdulescu GL, et al. The effects of social status on biological aging as measured by white-blood-cell telomere length. Aging Cell. 2006;5: 361–5. doi: 10.1111/j.1474-9726.2006.00222.x 16856882

[20] Needham BL, Rehkopf D, Adler N, Gregorich S, Lin J, Blackburn EH, et al. Leukocyte Telomere Length and Mortality in the National Health and Nutrition Examination Survey, 1999–2002: Epidemiology. 2015;26: 528–535. doi: 10.1097/EDE.0000000000000299 26039272 PMC4679150

[21] Pearson JA, Geronimus AT. A Practical Guide to Biological Primary Data Collection in an Impoverished Urban Setting: Illuminating Structural and Social Influences on Population Health Inequity. 1 Oliver’s Yard, 55 City Road, London EC1Y 1SP United Kingdom: SAGE Publications Ltd; 2018. doi: 10.4135/9781526444554

[22] Faul JD, Mitchell CM, Smith JA, Zhao W. Estimating telomere length heritability in an unrelated sample of adults: Is heritability of telomere length modified by life course socioeconomic status? Biodemography Soc Biol. 2016;62: 73–86. doi: 10.1080/19485565.2015.1120645 27050034 PMC5117361

[23] Kemp BR, Ferraro KF. Are Biological Consequences of Childhood Exposures Detectable in Telomere Length Decades Later? Le Couteur D, editor. The Journals of Gerontology: Series A. 2021;76: 7–14. doi: 10.1093/gerona/glaa019 31956916 PMC8355457

[24] Lapham K, Kvale MN, Lin J, Connell S, Croen LA, Dispensa BP, et al. Automated assay of telomere length measurement and informatics for 100,000 subjects in the genetic epidemiology research on adult health and aging (GERA) cohort. Genetics. 2015;200: 1061–1072. doi: 10.1534/genetics.115.178624 26092717 PMC4574243

[25] Lincoln KD, Lloyd DA, Nguyen AW. Social Relationships and Salivary Telomere Length Among Middle-Aged and Older African American and White Adults. Carr D, editor. The Journals of Gerontology: Series B. 2019;74: 1053–1061. doi: 10.1093/geronb/gbx049 28486613 PMC6703231

[26] Mitchell C, McLanahan S, Schneper L, Garfinkel I, Brooks-Gunn J, Notterman D. Father loss and child telomere length. Pediatrics. 2017;140. doi: 10.1542/peds.2016-3245 28716823 PMC5527665

[27] Rej PH, Bondy MH, Lin J, Prather AA, Kohrt BA, Worthman CM, et al. Telomere length analysis from minimally-invasively collected samples: Methods development and meta-analysis of the validity of different sampling techniques: American Journal of Human Biology. Am J Hum Biol. 2020; e23410. doi: 10.1002/ajhb.23410 32189404 PMC8105084

[28] Stout SA, Lin J, Hernandez N, Davis EP, Blackburn E, Carroll JE, et al. Validation of minimally-invasive sample collection methods for measurement of telomere length. Front Aging Neurosci. 2017/12/06 ed. 2017;9: 397. doi: 10.3389/fnagi.2017.00397 29270121 PMC5723637

[29] Zanet DL, Saberi S, Oliveira L, Sattha B, Gadawski I, Côté HCF. Blood and dried blood spot telomere length measurement by qPCR: assay considerations. PLoS One. 2013;8: e57787. doi: 10.1371/journal.pone.0057787 23451268 PMC3581490

[30] McDade TW, Stallings JF, Angold A, Costello EJ, Burleson M, Cacioppo JT, et al. Epstein-Barr Virus Antibodies in Whole Blood Spots: A Minimally Invasive Method for Assessing an Aspect of Cell-Mediated Immunity: Psychosom Med. 2000;62: 560–568. doi: 10.1097/00006842-200007000-00015 10949102

[31] McDade TW, Burhop J, Dohnal J. High-Sensitivity Enzyme Immunoassay for C-Reactive Protein in Dried Blood Spots. Clin Chem. 2004;50: 652. doi: 10.1373/clinchem.2003.029488 14981035

[32] McDade TW, Williams SA (Sharon AA, Snodgrass JJosh. What a Drop Can Do: Dried Blood Spots as a Minimally Invasive Method for Integrating Biomarkers Into Population-Based Research. Demography. 2007;44: 899–925. doi: 10.1353/dem.2007.0038 18232218

[33] Mitchell C, Hobcraft J, McLanahan SS, Siegel SR, Berg A, Brooks-Gunn J, et al. Social disadvantage, genetic sensitivity, and children’s telomere length. Proc Natl Acad Sci USA. 2014;111: 5944–5949. doi: 10.1073/pnas.1404293111 24711381 PMC4000782

[34] Goldman EA, Eick GN, Compton D, Kowal P, Snodgrass JJ, Eisenberg DTA, et al. Evaluating minimally invasive sample collection methods for telomere length measurement. Am J Hum Biol. 2018;30. doi: 10.1002/ajhb.23062 28949426 PMC5785450

[35] Demanelis K, Jasmine F, Chen LS, Chernoff M, Tong L, Delgado D, et al. Determinants of telomere length across human tissues. Science. 2020;369. doi: 10.1126/science.aaz6876 32913074 PMC8108546

[36] Astuti Y, Wardhana A, Watkins J, Wulaningsih W, PILAR Research Network. Cigarette smoking and telomere length: A systematic review of 84 studies and meta-analysis. Environ Res. 2017/07/10 ed. 2017;158: 480–489. doi: 10.1016/j.envres.2017.06.038 28704792 PMC5562268

[37] Espín-Pérez A, Portier C, Chadeau-Hyam M, van Veldhoven K, Kleinjans JCS, de Kok TMCM. Comparison of statistical methods and the use of quality control samples for batch effect correction in human transcriptome data. PLOS ONE. 2018;13: e0202947. doi: 10.1371/journal.pone.0202947 30161168 PMC6117018

[38] Zhang W-X, Fan J, Ma J, Rao Y-S, Zhang L, Yan Y-E. Selection of Suitable Reference Genes for Quantitative Real-Time PCR Normalization in Three Types of Rat Adipose Tissue. Int J Mol Sci. 2016;17. doi: 10.3390/ijms17060968 27338366 PMC4926500

[39] White H. A Heteroskedasticity-Consistent Covariance Matrix Estimator and a Direct Test for Heteroskedasticity. Econometrica. 1980;48: 817. doi: 10.2307/1912934

[40] Cunningham JM, Johnson RA, Litzelman K, Skinner HG, Seo S, Engelman CD, et al. Telomere length varies by DNA extraction method: implications for epidemiologic research. Cancer Epidemiol Biomarkers Prev. 2013;22: 2047–2054. doi: 10.1158/1055-9965.EPI-13-0409 24019396 PMC3827976

[41] Dagnall CL, Hicks B, Teshome K, Hutchinson AA, Gadalla SM, Khincha PP, et al. Effect of pre-analytic variables on the reproducibility of qPCR relative telomere length measurement. PLoS One. 2017/09/08 ed. 2017;12: e0184098. doi: 10.1371/journal.pone.0184098 28886139 PMC5590866

[42] Denham J, Marques FZ, Charchar FJ. Leukocyte telomere length variation due to DNA extraction method. BMC Res Notes. 2014;7: 877. doi: 10.1186/1756-0500-7-877 25475541 PMC4289347

[43] Hofmann JN, Hutchinson AA, Cawthon R, Liu C-S, Lynch SM, Lan Q, et al. Telomere length varies by DNA extraction method: implications for epidemiologic research-letter. Cancer Epidemiol Biomarkers Prev. 2014;23: 1129–1130. doi: 10.1158/1055-9965.EPI-14-0145 24798729 PMC4051398

[44] Lahiri DK, Nurnberger JI. A rapid non-enzymatic method for the preparation of HMW DNA from blood for RFLP studies. Nucleic Acids Res. 1991;19: 5444. doi: 10.1093/nar/19.19.5444 1681511 PMC328920

[45] D’haene B, Vandesompele J, Hellemans J. Accurate and objective copy number profiling using real-time quantitative PCR. Methods. 2010;50: 262–270. doi: 10.1016/j.ymeth.2009.12.007 20060046

[46] Geronimus AT. Deep Integration: Letting the Epigenome Out of the Bottle Without Losing Sight of the Structural Origins of Population Health. Am J Public Health. 2013;103: S56–S63. doi: 10.2105/AJPH.2013.301380 23927509 PMC3786760

