# Supplementary material for: Coming up short: Comparing venous blood, dried blood spots & saliva samples for measuring telomere length in health equity research
Source: PLoS One. 2021 Aug 18;16(8):e0255237. doi: 10.1371/journal.pone.0255237 (PMC8372921; doi:10.1371/journal.pone.0255237)
Supplement: S1 Appendix — (DOCX) [file pone.0255237.s001.docx]

**S1 Appendix**

**Supplementary Information**

**Coming Up Short: Comparing Venous Blood, Dried Blood Spots & Saliva Samples for Measuring Telomere Length**

***Sample Recruitment***

We recruited a total of 132 non-Hispanic Black, non-Hispanic White, and Mexican-descent Michigan women residents from disinvested high-poverty areas of Detroit and a more affluent area, Ann Arbor, ages 25-49. Telomere length (TL) was measured in DNA from three distinct specimen types from each woman: fresh blood (PBMC), dried blood spot (DBS), and saliva.

By including Detroit residents of high poverty areas, we simulate a target sample that includes members of marginalized groups. We focused on women as previous research has shown women to suffer the steepest age-gradient increase and the largest racial/ethnic differences in chronic disease and disability.^1,2^ In addition, there is some evidence that the ways in which adverse childhood experience influences adult telomere length varies between men and women.^3^ We chose the age range of 25-49 to encompass ages unlikely to be either too young or too old to detect TL differences unrelated to normal biological growth, development, or aging, and ages with less potential to be affected by survival bias than older ages. Previous social epidemiological studies of population differences in stress-mediated health and aging find these are the ages where structurally patterned population health inequities in TL diverge importantly.^2,4^ While generally the rate of telomere shortening in this age group is small relative to younger and older ages, the range is large^5,6^ and might be associated with socially patterned biopsychosocial, behavioral, or environmental inputs. In addition, limiting survival bias is a critically important concern when studying population health inequity where variations in life expectancy distributions are marked and likely associated with the same fundamental social causes as any differences in TL, diluting or distorting disparities between populations.^1,5,7^ We note that people sampled in several previous studies detecting an impact of social stressors on TL were in their late 30s, on average.^8–10^

Detroit, MI participants were recruited through paper flyers we distributed across the city (bus stops, beauty shops, laundromats, grocery markets, apartment complexes) and at community events (swap meets, community yard sales, sports events and concerts, and summer festivals), as well as on social media (e.g., Facebook community groups and non-profit organization sites specific to Detroit). We recruited participants from the Ann Arbor, MI area using a university-administered website, UM Clinical Studies, which connects potential volunteers with researchers. Additionally, we posted flyers across campus inviting appropriately aged women who self-identify as either white, African American, or Latina to contact our study personnel for further information. The flyer, written in both English and Spanish, described the study and a telephone number for more information. Participants were also referred by word-of-mouth in both Detroit and Ann Arbor. Note: We were able to analyze a convenience sample because our primary scientific question is whether *within-woman* TL differences found in fresh blood cells compared to DBS or to saliva are systematic or random with respect to age, race/ethnicity, education, or residential area − and not whether there are racial/ethnic or other differences in TL, *per se.*

***Details of Study Appointment and Data Collection***

When potential participants called for more information, our project staff described the study purpose and protocol, completed a standardized screener brief intake to assess participant eligibility, answered questions and then scheduled an appointment if they agreed to participate in the study. For complete survey data, blood pressure measurement, and biospecimen collection, participants were offered $100 remuneration to thank them for their time and information. Participants were also provided with their blood pressure measurements and a recommendation for follow-up with a health care provider when appropriate. The protocol was IRB approved.

At the start of each scheduled visit, the study purpose, protocol, and consent were described, and any additional questions were answered by the research staff in the preferred language of the participant (English or Spanish). After obtaining written informed consent, the visit continued in the preferred language of the participant and research staff measured the participants’ resting, seated systolic and diastolic blood pressures (mmHg) using a factory calibrated electronic oscillometric blood pressure monitor and appropriately sized arm cuff. Next, we administered a brief survey to elicit background information, and to identify any contraindications/preclusions to venous blood draw in particular participants (e.g., very high blood pressure or usage of certain medications). 11 participants with two failed blood draws were excluded from the final analytic sample but compensated for their participation. The appointments generally lasted 60-90 minutes.

Three diverse teams recruited research participants, obtained informed consent, and collected their data. Each team included a team leader, their assistant, and a phlebotomist; each team included a native Spanish speaker. The teams were led by a Mexican-descent woman, an African American Women, and an Arab-American woman, representing the three major racial/ethnic groups in Detroit. The phlebotomists were current Detroit residents, a Black male, a Black female, and a White female. Team assistants included one Mexican–descent female and one White male. Participants chose whether they preferred to have the data collection visit conducted in English or Spanish and, in Detroit, whether they preferred to have their specimens collected through a home visit or at the University of Michigan Detroit Center, a UM building in Detroit that accommodates research projects and outreach initiatives. Data collection in Ann Arbor occurred in a private room at the University of Michigan Institute for Social Research. After collection, all specimens were prepared for shipment in the same lab and by the same technician at the University of Michigan; after shipment all were analyzed in the same lab in Princeton NJ. To minimize batch effects when measuring telomere length, all samples from the same individual were included on the same qPCR run***.***

Interviewers used an Oragene® OGR -500 kit (DNA Genotek, Ottawa, Ontario) for saliva sample collection, following protocols to collect approximately 2 ml of saliva from each participant. Four 10-ml tubes of peripheral venous blood were collected in sterile endotoxin-free vacutainers with anticoagulant by a single venipuncture by a professional phlebotomist using standard techniques. The phlebotomist also collected whole blood via DBS, using a routine protocol for collecting 50 μL generated by pricking the finger with a small sterile lancet, letting blood drops fall onto and dry on filter paper and storing samples in plastic bags with a humidity indicator desiccant ^11^. Dried blood spots were collected on Whatman Protein Saver 903 cards (GE Healthcare, Chicago, IL) and shipped to the lab at room temperature.

***Specimen Collection* *& Genomic DNA Isolation***

Within 6 hours of collection, peripheral blood mononuclear cells were isolated by the Histopaque‐1077 (Sigma-Aldrich, St. Louis) density‐gradient method from blood, aliquoted, and stored at -80C, until two aliquots were sent to the lab on dry ice overnight in accordance with domestic biological shipping regulations. The third aliquot of PBMCs was maintained as a reserve. All PBMC specimens were maintained in the lab at -80^o^C until use or the end of the project.

Saliva specimens were kept in a secure and locked location and maintained at room temperature until mailed to the same laboratory for analysis. The lab promptly extracted DNA from the saliva specimens using the Oragene® Laboratory Protocol Manual Purification of DNA (DNA Genotek, Ottawa, Ontario). DNA was extracted from PBMC cell pellets using the PureLink genomic DNA mini kit (Invitrogen, Carlsbad, CA), a silica-based membrane kit comparable to the Qiagen DNA blood mini kit^12^, according to the manufacturer’s protocol. Three dried blood spots were used for extraction using the PureLink genomic DNA mini kit according to the manufacturer’s protocol. All samples purified using the PureLink kit were eluted with elution buffer (10 mM Tris-HCl, pH 9.0, 0.1 mM EDTA). DNA concentration was determined using the Quant-iT PicoGreen dsDNA Assay Kit (Thermo Fisher Scientific, Waltham, MA) and stored at -80 °C. The average DNA concentrations from DBS, saliva and PBMC samples were 5.4 ng/μL, 104.4 ng/μL and 74.1 ng/μL, respectively.

***Telomere Length (TL) Measurement***

The lab measured TL using a qPCR assay that incorporated an oligomer standard to permit measurement of absolute (in kilobases per telomere) rather than relative TL.^13–15^ Briefly, this method adapts the approach of Cawthon^16^ in which relative TL is determined by qPCR by determining the ratio of telomere copy repeats to a single copy reference gene (*36B4*, which encodes acidic ribosomal phosphoprotein P0). To determine absolute TL, an 84mer double stranded oligomer standard containing telomere repeats ((5’-TTAGGG-3’)_14_ and its complement) was used to construct a standard curve.  A separate standard curve for the single copy gene incorporates a 79mer double stranded oligonucleotide containing sequence from *36B4* (5’-TTC AGC AAG TGG GAA GGT GTA ATC CGT CTC CAC AGA CAA GGC CAG GAC TCG TTT GTA CCC GTT GAT GAT AGA GTG GGG T-3’ and its complement). This enables calculation of total TL per diploid genome, while the 36B4 product gives the number of diploid genomes. Samples (3ng/well) were assayed separately for telomere (tel) and 36B4 quantity in triplicate and the results averaged. If the standard deviation of the triplicate Ct was > 0.34, the outlier was removed prior to data analysis. This was also done for the standards and controls run on each plate. The tel qPCR reaction (1x QuantiTect SYBRGreen (Qiagen, Hilden, Germany), teloF (5’-CGG TTT GTT TGG GTT TGG GTT TGG GTT TGG GTT TGG GTT-3’) and teloR (5’-GGC TTG CCT TAC CCT TAC CCT TAC CCT TAC CCT TAC CCT-3’) primers (0.3 μM each^17^), Uracil N-Glycosylase (UNG; 0.2 U; Thermo Fisher Scientific, Waltham, MA)) was run using on an Applied Biosystems QuantStudio 6 Flex Machine using the following cycling conditions: 1 cycle: 2 minutes at 50 °C, 1 cycle: 15 minutes at 95 °C, 40 cycles: 15 seconds at 95 °C and 1 minute at 60 °C. The *36B4* qPCR reaction was the same except that 0.3 μM 36B4U (5’-CAG CAA GTG GGA AGG TGT AAT CC-3’) and 0.5 μM 36B4D (5’-CCC ATT CTA TCA TCA ACG GGT ACA A-3’) primers^17^ were used instead of teloF and teloR. All oligonucleotides were from Integrated DNA Technologies (IDT, Coralville, Iowa).

To minimize batch effects, all samples from the same individual were included on the same qPCR run. In addition to the appropriate standards, each 384-well plate contained two DNA samples, also in triplicate, that were repeated on each run to mitigate batch effects. These were: reference genomic DNA from a cell line with a relatively short telomere (3C167b), reference genomic DNA from a fibroblast cell line containing a stably integrated hTERT gene (NHFpreT)^18,19^. Reference genomic DNAs were harvested at a single time, aliquoted, and frozen. In addition, two DNA samples were purified from volunteer saliva, aliquoted and used by the lab to measure the variation between plates. Standards (with pBR322 plasmid DNA (0.5 ng/ul) added for stabilization), and primers were diluted to the appropriate concentrations and batch frozen in single use aliquots for the project. The geometric mean of the two cell lines’ (3C167b and NHFpreT) telomere quantities from each run was divided by the geometric mean of the two cell line telomere quantities from all the runs to create a normalization factor for each run. This procedure was repeated for the 36B4 quantities. TL was calculated using Eq. 1. The coefficients of variation the two volunteer DNAs were 10%. If using the average of the relative TL (T/S) of the two controls, the coefficient of variation was less than one percent. As elaborated below, we measured the reliability of our measures of ln(TL). In all cases the within-person variability of ln(TL) is extremely small relative to the between person variability, implying reliability coefficients of 0.98.

***Statistical Framework***

As discussed in the body of the paper, much of the interest in TL involves using measured TL as an indicator of what has been termed, “biological age.” It will be of some value to formalize this notion in terms of a simple statistical model. Let *y*_ij_ represent measured TL for individual i using specimen j (*y* = ln(TL)) and let *α*_i_ represent their biological age. We do not observe *α*_i_ but use *y*_ij_ as an indicator for this quantity:

*y*_ij_ = *α*_i_  + ν_ij_  + ε_ij_ (2)

ε_ij_ represents average test/retest error when measuring *y*, and can plausibly be assumed independent of *α*_i_ . ν_ij_  represents the gap between telomere length in the sample and the biological age of the individual. Telomere length is an indicator of biological age, not biological age itself. The fact that telomere length is measured in triplicate gives us a way to measure var(ε_ij_). If we assume that *α*_i_ . and ν_ij_  are independent of each other, we can use the variance covariance matrix across specimen types to identify var(*α*_i_ ) and var(ν_ij_ ) in our samples.

What researchers are typically interested in is the association between biological age (conditional on chronological age) and various aspects of individuals’ socially structured experience. Thus, for example, researchers have been interested in the effects of stress on telomere length^8,9^ in the association between race and telomere length^4^, and in the effect of early disadvantage on telomere length.^13^ Thinking of TL as an indicator of biological age, the regression that these researchers would have liked to run would have been:

*α*_i_  = *X*_i_’β + *μ*_i_, (3)

where *X* include both variables of particular interest and controls. We will assume that *μ* is orthogonal to *X*. The motivation for this assumption is strategic. We want to focus on the impact that using TL as an indicator for biological age has on our estimates. If we could observe *α*_i_  ols estimates of (3) would yield unbiased estimates of β. Without direct measures of α, the researchers use *y* as an indicator of α, and regresses *y* on *X*.

*y*_ij_ = *X*_i_’β + *μ*_i_, + ν_ij_  + ε_ij_ (4)

Letting *b* represent the projection of *y*_ij_ on *X*_i_,

*b* = (*X_i_’X_i_*)^-1^*X_i_’y*_ij_ = (*X*_i_*’X*_i_)^-1^*X*_i_*’*(*X*_i_’β + *μ*_i_,+ ν_ij_  + ε_ij_) (5)

Given the assumptions we have made about μ and ε,

E(*b*) = β + (*X*_i_*’X*_i_)^-1^*X*_i_*’* ν_ij_ . (6)

If, as researchers have presumed, ν is “random noise” and independent of *X*, then OLS estimates of *b* represent unbiased estimates of β. However, if there is a systematic component to ν, with the implication that *E*(ν_i_|*X*_i_) ≠ 0, then *b* will be biased. We cannot test *E*(ν_i_|*X*_i_) = 0 directly. However, we can compare estimates of *b* across specimen types. If *E*(ν_i_|*X*_i_) = 0 for all specimen types then estimates of *b* should not vary significantly across specimen types. If, on the other hand, estimates of *b* vary significantly, this suggest that *E*(ν_i_|*X*_i_) ≠ 0 for at least some specimen types.

With this framework in mind, we regressed our ln(TL) measures on the age of our respondents, dummies representing their race and ethnicity, their educational attainment and a location dummy (Ann Arbor vs Detroit). Since our samples are convenience samples and do not follow individuals over time, one cannot interpret the coefficients as reflecting cross group differences for anything but this sample. However, if TL measures based on the different tissues are all valid measures of biological age (in our model if *E*(ν_i_|*X*_i_) = 0) we would expect the association between age, race, place or education and TL to be the same across these different specimens within the precision of the assay.

***Reliability of Measures***

TL was measured in DNA extracted from three the distinct specimen types: fresh blood (PBMC), dried blood spot (DBS), and saliva. Telomere and 36B4 quantities were interpolated from the standard curve generated from the reference fragments of known length. All samples were divided into three technical replicates, which were averaged in the following formula:

ln(TL) = [(Avg. ln(Tel qty) – ln(Tel normalization factor))] – [(Avg. ln(36B4 Qty)) – ln(36B4 normalization factor))] – ln(92) (1)

Note that (1) represents ln(TL) as linear in terms. This turns out to be useful from the point of view of our statistical analysis. The purpose of using triplicate measurement of the same sample is to reduce random measurement error. In our case, we also used these separate measurements to measure the magnitude of the “noise” when measuring TL. This “noise” could be due to either imprecise measurement or to sampling variation in TL quantity. In particular, for each individual in each of our specimens, we have three separate measures of ln(Tel qty) and ln(36B4 Qty) roughly 75% of the time (see Table 2). For the remaining 25%, we have two separate measures.

When measuring Avg. ln(Tel qty) and Avg. ln(36B4 Qty) we are interested in the statistical population mean of these quantities, or the average we would obtain were the lab to make these measurements for every possible cell within the biological sample. However, what we have for each person measurements of three or, sometimes two, draws from the biological sample. We are using these sample averages as estimates for the population averages in which we are interested. If we assume that individual measurements are random draws from the universe of possible measurements on this sample, we can use simple statistics to derive the sampling distribution of our estimates. It should be noted that this procedure allows us to estimate the amount of test-retest error in our measures.

If we think of the within-person sample averages of ln(Tel qty) and ln(36B4 Qty) as estimates of population averages of these quantities, we can use the within-person variation in ln(Tel qty) and ln(36B4 Qty) to gauge the within-batch reliability of our measurements of ln(Tel qty) and ln(36B4 Qty). If there are measurement effects, or there are sample effects in the sense that blood samples from an individual today might different from samples from the same person on another occasion, we will not be able to detect these.

As discussed above, we can use the fact that we typically have three measures of ln(Tel qty) and ln(36B4 Qty)) to measure the reliability of our measures of ln(TL). Table S1 reports the average within-person variation in ln(Tel qty) and ln(36B4 Qty). We ran all samples for an individual in the same batch to reduce possible confounds related to batch effects.

Using the average within person sample variance in ln(Tel qty) and ln(36B4 Qty) as our measures of variability, we see that the average variance for ln(Tel qty) range from 0.005 to 0.007, while the average variance for ln(36B4 Qty) ranges from 0.003 to 0.004. The lower ranges of 36B4 Qty variance provide an estimate of the measurement error lower bound of this method. Further that TL Qty has only slightly higher variance implies that the majority of the within person variance is due to measurement error and not sampling variation in TL aliquots. Nevertheless, both have very low levels of measurement error. We can use these estimates to assess the reliability of the measures reported in Table 3. Table S2 reports our estimates of the reliability of each of our measures of ln(TL). In all cases the within-person variability of ln(TL) is extremely small relative to the between person variability, implying reliability coefficients around 0.98.

One clear implication of the reliability coefficients being so high is that correcting for measurement error would have very little effect on our estimates of the correlation between our ln(TL) measures based on the different specimens.

Table S1. Mean within person variance by specimen type

|  |  | ln(Tel Quant) | ln(36B4 Quant) |
| --- | --- | --- | --- |
| Full Sample | PBMC | 0.0059 | 0.0031 |
|  | Saliva | 0.0045 | 0.0034 |
| DBS complete sample | PBMC | 0.0062 | 0.0029 |
|  | Saliva | 0.0051 | 0.0032 |
|  | DBS | 0.0067 | 0.0041 |

Table S2. Mean variance and reliability estimates by specimen type

|  |  | (1/n^2 x var(ln(tel qty))) | (1/n^2 x var(ln(36B4 Quant))) | Reliability Estimate |
| --- | --- | --- | --- | --- |
| Full Sample | PBMC | 0.00077 | 0.00038 | 0.98 |
|  | Saliva | 0.00059 | 0.00039 | 0.98 |
| DBS complete sample | PBMC | 0.00081 | 0.00034 | 0.98 |
|  | Saliva | 0.00066 | 0.00036 | 0.98 |
|  | DBS | 0.00082 | 0.00046 | 0.98 |

***Robustness***

We did an analysis of our samples to confirm that our results were robust to the exclusion of outliers. Our primary interest in this work was to understand whether regression results were dependent the choice of tissue collected. With this in mind, we checked the regressions that used differences in measured telomere length as measured using the different tissue types as our dependent variables. Table S3 shows the distribution of Cook’s D statistic^20^ for our two samples. Using critical cuts offs of 0.03 for the larger sample and 0.04 for the smaller sample, we found 11 influential observations in the larger sample, and either 11 or 12 influential observations in the smaller sample.

Table S3. Distributions of Cook's D

|  | Full Sample | | | DBS Complete Sample | | | | |
| --- | --- | --- | --- | --- | --- | --- | --- | --- |
| Percentile | PBMC | Saliva | Saliva-PBMC | PBMC | Saliva | Saliva-PBMC | DBS | DBS-PBMC |
|  |  |  |  |  |  |  |  |  |
| 5% | 0.000 | 0.000 | 0.000 | 0.000 | 0.000 | 0.000 | 0.000 | 0.000 |
| 25% | 0.001 | 0.001 | 0.001 | 0.001 | 0.001 | 0.001 | 0.002 | 0.001 |
| 50% | 0.003 | 0.003 | 0.003 | 0.003 | 0.005 | 0.004 | 0.004 | 0.004 |
| 75% | 0.009 | 0.010 | 0.008 | 0.010 | 0.013 | 0.011 | 0.010 | 0.013 |
| 95% | 0.039 | 0.036 | 0.029 | 0.052 | 0.047 | 0.045 | 0.039 | 0.050 |
| cutoff | 0.03 | 0.03 | 0.03 | 0.04 | 0.04 | 0.04 | 0.04 | 0.04 |

Table S4 shows results from eliminating these influential observations. As the estimates show, eliminating these observations had little effect on our conclusions that in these samples the specimen used affects conclusions about the association between the variables in our analysis and measured telomere length.

Table S4. Comparing results after removing outliers.

|  | Full Sample | | DBS Complete Sample | | | |
| --- | --- | --- | --- | --- | --- | --- |
|  | Original | Trimmed | Original | Trimmed | Original | Trimmed |
| Variable | Saliva-PBMC | Saliva-PBMC | Saliva-PBMC | Saliva-PBMC | DBS-PBMC | DBS-PBMC |
| Age/10 | 0.067 | 0.093 | 0.038 | 0.039 | 0.023 | 0.021 |
|  | (0.022) | (0.018) | (0.028) | (0.019) | (0.036) | (0.024) |
| Detroit | 0.036 | -0.019 | 0.060 | 0.012 | -0.020 | -0.087 |
|  | (0.042) | (0.028) | (0.048) | (0.029) | (0.061) | (0.040) |
| Black | 0.102 | 0.023 | 0.104 | 0.051 | 0.092 | -0.021 |
|  | (0.040) | (0.031) | (0.047) | (0.036) | (0.056) | (0.042) |
| Hispanic | 0.020 | 0.016 | 0.001 | 0.001 | 0.019 | -0.036 |
|  | (0.039) | 0.032 | (0.045) | (0.035) | (0.056) | (0.039) |
| High School | -0.001 | 0.112 | -0.006 | 0.062 | -0.203 | -0.144 |
|  | (0.049) | (0.039) | (0.053) | (0.043) | (0.059) | (0.049) |
| College and Above | -0.011 | 0.105 | -0.042 | 0.038 | -0.134 | -0.080 |
|  | (0.053) | (0.042) | (0.056) | (0.044) | (0.064) | (0.049) |
| Joint Test | 0.004 | 0.0001 | 0.086 | 0.055 | 0.012 | 0.0085 |
| n | 132 | 121 | 99 | 87 | 99 | 88 |

*Robust standard errors in parenthesis ^21^

***Heparinase Treatment of Samples***

Heparin is a known inhibitor of DNA polymerases including Taq DNA polymerase, which is used in the qPCR measurement of telomere length.^22–24^ Heparinase can reverse this inhibition.^23,25–27^ Since blood was collected in sodium heparin vacutainer tubes, it was possible that residual heparin in the sample affected the TL measurement by qPCR. Also, the degree of the effect of heparin can vary, depending upon the source of Taq.^24^

The effect of the use of heparin as an anticoagulant on TL qPCR measurement has not been extensively characterized. Stout et al.^28^ used venous blood collected in heparin vacutainer tubes and found a high correlation (*r* = 0.84, p<0.001) between relative TL in DBS and whole blood and reasonable correlation between relative TL in saliva and whole blood (r = 0.56, p = 0.005). At least one study found no significant difference in TL from blood collected in EDTA, acid citrate dextrose or heparin vacutainer tubes.^29^

We confirmed experimentally that the TL measurements in these samples were ***not*** affected by the use of heparin as the anticoagulant. Samples from the original study were retested with and without the addition of heparinase. If there were a meaningful heparin effect on the measurement in the PMBC samples, then this should have been reversed by the addition of heparinase. Genomic DNA (1.5 ug purified from saliva or PBMCs isolated from sodium heparin tubes) was treated with 12 U of *Bacteroides* Heparinase I (New England Biolabs) for 2 hours at room temperature. The control contained 1.5 ug of genomic DNA treated identically (similarly diluted, incubated with heparinase buffer for 2 hours at room temperature) with the exception that heparinase was not added. TL was measured in triplicate twice using 1.5 ng genomic DNA /well as described in the manuscript except that values were not normalized for variation between runs. The averaged results across the two experiments are shown in SI Figure 1. SI Figure 1 C shows the effect on TL of adding heparinase. No PMBC sample had a reproducibly higher TL after heparin treatment. Also, the telomere quantities (Fig. 1A) and 36B4 (Fig. 1B) quantities of the PBMC samples assayed were not reproducibly higher in the presence of heparinase *indicating that heparin is not a significant confounder in these samples.*


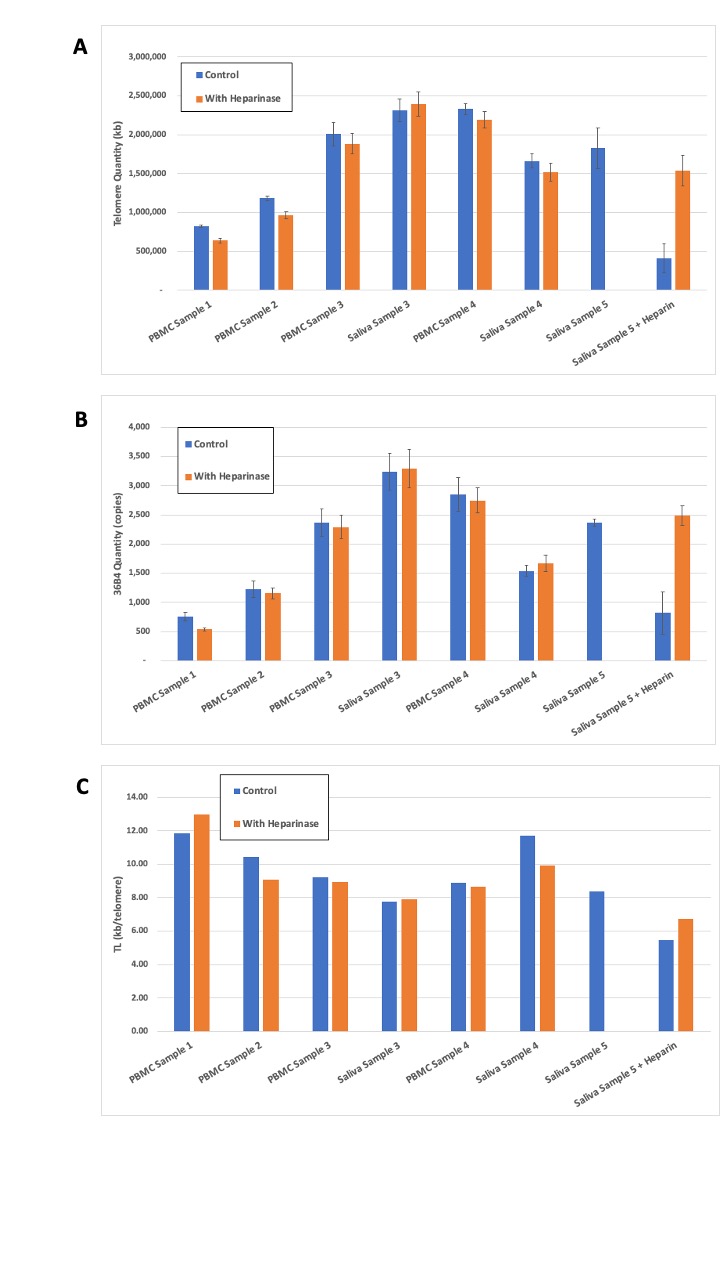


SI Figure 1: The effect of pretreatment of PBMC and saliva samples prior to telomere length measurement by qPCR. Samples were incubated for two hours in the absence or presence of heparinase at room temperature. TL was measured by qPCR. Samples were measured in triplicate and the qPCR repeated once. Bars represent average values from each qPCR run; error bars represent the standard error. The sample number refers to an individual (e.g. PBMC Sample 4 and Saliva Sample 4 represent genomic DNA purified from blood and saliva from the same individual). Heparin was added to Saliva sample 5 (the same sample as in Figure 2) as a control. A. qPCR reaction measuring telomere quantity. B. qPCR reaction measuring 36B4 quantity. C. TL measurement.

**Supplemental Information References**

1. Geronimus AT, Bound J, Waidmann TA, Colen CG, Steffick D. Inequality in life expectancy, functional status, and active life expectancy across selected black and white populations in the United States. *Demography*. 2001;38(2):227-251. doi:10.1353/dem.2001.0015

2. Geronimus AT, Hicken M, Keene D, Bound J. “Weathering” and Age Patterns of Allostatic Load Scores Among Blacks and Whites in the United States. *Am J Public Health*. 2006;96(5):826-833. doi:10.2105/AJPH.2004.060749

3. Kemp BR, Ferraro KF. Are Biological Consequences of Childhood Exposures Detectable in Telomere Length Decades Later? Le Couteur D, ed. *The Journals of Gerontology: Series A*. 2021;76(1):7-14. doi:10.1093/gerona/glaa019

4. Geronimus AT, Pearson JA, Linnenbringer E, et al. Race-Ethnicity, Poverty, Urban Stressors, and Telomere Length in a Detroit Community-based Sample. *Journal of Health and Social Behavior*. 2015;56(2):199-224. doi:10.1177/0022146515582100

5. Frenck RW, Blackburn EH, Shannon KM. The rate of telomere sequence loss in human leukocytes varies with age. *Proceedings of the National Academy of Sciences*. 1998;95(10):5607-5610. doi:10.1073/pnas.95.10.5607

6. Aviv A. The Epidemiology of Human Telomeres: Faults and Promises. *The Journals of Gerontology Series A: Biological Sciences and Medical Sciences*. 2008;63(9):979-983. doi:10.1093/gerona/63.9.979

7. Geronimus AT, Bound J, Waidmann TA, Rodriguez JM, Timpe B. Weathering, Drugs, and Whack-a-Mole: Fundamental and Proximate Causes of Widening Educational Inequity in U.S. Life Expectancy by Sex and Race, 1990–2015. *J Health Soc Behav*. 2019;60(2):222-239. doi:10.1177/0022146519849932

8. Cherkas LF, Aviv A, Valdes AM, et al. The effects of social status on biological aging as measured by white-blood-cell telomere length. *Aging Cell*. 2006;5(5):361-365. doi:10.1111/j.1474-9726.2006.00222.x

9. Epel ES, Blackburn EH, Lin J, et al. Accelerated telomere shortening in response to life stress. *Proc Natl Acad Sci U S A*. 2004;101(49):17312-17315. doi:10.1073/pnas.0407162101

10. Lapham K, Kvale MN, Lin J, et al. Automated assay of telomere length measurement and informatics for 100,000 subjects in the genetic epidemiology research on adult health and aging (GERA) cohort. *Genetics*. 2015;200(4):1061-1072. doi:10.1534/genetics.115.178624

11. McDade TW, Williams SA (Sharon AA, Snodgrass JJosh. What a Drop Can Do: Dried Blood Spots as a Minimally Invasive Method for Integrating Biomarkers Into Population-Based Research. *Demography*. 2007;44(4):899-925. doi:10.1353/dem.2007.0038

12. Denham J, Marques FZ, Charchar FJ. Leukocyte telomere length variation due to DNA extraction method. *BMC Res Notes*. 2014;7:877. doi:10.1186/1756-0500-7-877

13. Mitchell C, Hobcraft J, McLanahan SS, et al. Social disadvantage, genetic sensitivity, and children’s telomere length. *Proceedings of the National Academy of Sciences*. 2014;111(16):5944-5949. doi:10.1073/pnas.1404293111

14. Mitchell C, McLanahan S, Schneper L, Garfinkel I, Brooks-Gunn J, Notterman D. Father loss and child telomere length. *Pediatrics*. 2017;140(2). doi:10.1542/peds.2016-3245

15. O’Callaghan NJ, Fenech M. A quantitative PCR method for measuring absolute telomere length. *Biol Proced Online*. 2011;13:3. doi:10.1186/1480-9222-13-3

16. Cawthon RM. Telomere measurement by quantitative PCR. *Nucleic Acids Research*. 2002;30(10):47e-447. doi:10.1093/nar/30.10.e47

17. O’Callaghan NJ, Dhillon VS, Thomas P, Fenech M. A quantitative real-time PCR method for absolute telomere length. *BioTechniques*. 2008;44(6):807-809. doi:10.2144/000112761

18. Cheng, D., Zhao Y, Wang S, et al. Repression of telomerase gene promoter requires human-specific genomic context and is mediated by multiple HDAC1-containing corepressor complexes. *FASEB J*. 2017;31(3):1165-1178. doi:10.1096/fj.201601111R

19. Wang S, Zhu J. Evidence for a relief of repression mechanism for activation of the human telomerase reverse transcriptase promoter. *J Biol Chem*. 2003;278(21):18842-18850. doi:10.1074/jbc.M209544200

20. Cook RD. Influential Observations in Linear Regression. *Journal of the American Statistical Association*. 1979;74(365):169-174. doi:10.1080/01621459.1979.10481634

21. White H. A Heteroskedasticity-Consistent Covariance Matrix Estimator and a Direct Test for Heteroskedasticity. *Econometrica*. 1980;48(4):817. doi:10.2307/1912934

22. Holodniy M, Kim S, Katzenstein D, Konrad M, Groves E, Merigan TC. Inhibition of human immunodeficiency virus gene amplification by heparin. *J Clin Microbiol*. 1991;29(4):676-679. doi:10.1128/JCM.29.4.676-679.1991

23. Izraeli S, Pfleiderer C, Lion T. Detection of gene expression by PCR amplification of RNA derived from frozen heparinized whole blood. *Nucleic Acids Res*. 1991;19(21):6051. doi:10.1093/nar/19.21.6051

24. Yokota M, Tatsumi N, Nathalang O, Yamada T, Tsuda I. Effects of heparin on polymerase chain reaction for blood white cells. *J Clin Lab Anal*. 1999;13(3):133-140. doi:10.1002/(sici)1098-2825(1999)13:3<133::aid-jcla8>3.0.co;2-0

25. Johnson ML, Navanukraw C, Grazul-Bilska AT, Reynolds LP, Redmer DA. Heparinase treatment of RNA before quantitative real-time RT-PCR. *Biotechniques*. 2003;35(6):1140-1142, 1144. doi:10.2144/03356bm03

26. Kondratov K, Kurapeev D, Popov M, et al. Heparinase treatment of heparin-contaminated plasma from coronary artery bypass grafting patients enables reliable quantification of microRNAs. *Biomol Detect Quantif*. 2016;8:9-14. doi:10.1016/j.bdq.2016.03.001

27. Roest HP, Verhoeven CJ, de Haan JE, de Jonge J, IJzermans JNM, van der Laan LJW. Improving Accuracy of Urinary miRNA Quantification in Heparinized Patients Using Heparinase I Digestion. *J Mol Diagn*. 2016;18(6):825-833. doi:10.1016/j.jmoldx.2016.06.006

28. Stout SA, Lin J, Hernandez N, et al. Validation of minimally-invasive sample collection methods for measurement of telomere length. *Front Aging Neurosci*. 2017;9:397. doi:10.3389/fnagi.2017.00397

29. Zanet DL, Saberi S, Oliveira L, Sattha B, Gadawski I, Côté HCF. Blood and dried blood spot telomere length measurement by qPCR: assay considerations. *PLoS One*. 2013;8(2):e57787. doi:10.1371/journal.pone.0057787
